# Tumor heterogeneity assessed by texture analysis on contrast-enhanced CT in lung adenocarcinoma: association with pathologic grade

**DOI:** 10.18632/oncotarget.15399

**Published:** 2017-02-16

**Authors:** Ying Liu, Shichang Liu, Fangyuan Qu, Qian Li, Runfen Cheng, Zhaoxiang Ye

**Affiliations:** ^1^ Department of Radiology, Tianjin Medical University Cancer Institute and Hospital, National Clinical Research Center of Cancer, Key Laboratory of Cancer Prevention and Therapy, Tianjin, China; ^2^ Department of Pathology, Tianjin Medical University Cancer Institute and Hospital, National Clinical Research Center of Cancer, Key Laboratory of Cancer Prevention and Therapy, Tianjin, China

**Keywords:** lung, adenocarcinoma, international association for the study of lung cancer/American thoracic society/European respiratory society, pathologic grade, heterogeneity

## Abstract

Objectives To investigate whether texture features on contrast-enhanced computed tomography (CECT) images of lung adenocarcinoma have association with pathologic grade.

Method*s* A cohort of 148 patients with surgically operated adenocarcinoma was retrospectively reviewed. Fifty-four CT features of the primary lung tumor were extracted from CECT images using open-source 3D Slicer software; meanwhile, enhancement homogeneity was evaluated by two radiologists using visual assessment. Multivariate logistic regression analysis was performed to determine significant image indicator of pathologic grade.

Results Tumors of intermediate grade were more likely to be never smokers (*P*=0.020). Enhancement heterogeneity by visual assessment showed no statistical difference between intermediate grade and high grade (*P*=0.671). Among those 54 features, 29 of them were significantly associated with pathologic grade. Multivariate logistic regression analyses identified F33 (Homogeneity 1) (*P*=0.005) and F38 (Inverse Variance) (*P*=0.032) as unique independent image indicators of pathologic grade, and the AUC calculated from this model (AUC=0.834) was higher than clinical model (AUC=0.615) (*P*=0.0001).

Conclusions Our study revealed that texture analysis on CECT images could be helpful in predicting pathologic grade of lung adenocarcinoma.

## INTRODUCTION

Lung cancer is the leading cause of cancer related death worldwide [1]. Non-small-cell lung cancer (NSCLC) accounts for 75-80 % of lung cancer, with adenocarcinomas being the most common histological subtype [2]. Lung adenocarcinoma is known as a heterogeneous tumor from every perspective, including molecular, clinical, radiological, surgical, and pathological aspects [3]. Acknowledging this, a new histologic classification system for lung adenocarcinomas was proposed in 2011 with the joint effort of International Association for the Study of Lung Cancer (IASLC), American Thoracic Society (ATS) and European Respiratory Society (ERS) [4]. The terminology and criteria of the new classification system are intended to better guide routine patient care and to improve accuracy of data collection for clinical trials [5].

One of the major changes of this new IASLC/ATS/ERS classification is that invasive adenocarcinoma subtypes are classified by predominant pattern after performing comprehensive histological subtyping with semiquantitative assessment of each subtype in 5% increments [4]. A remarkable correlation between survival and histopathological subtypes of adenocarcinoma on the basis of this new classification has been validated in several studies [3, 6–10]. Xu et al. [6] reported that patients with micropapillary and solid predominant tumors had a significantly worse overall survival and disease free survival as compared to those with other subtypes predominant tumors. In another study [9], it also confirmed that patients with micropapillary or solid predominant patterns had poor overall survival. Yoshizawa et al. [3] pointed out that patients of intermediate grade (lepidic/ acinar/papillary predominant) had significantly better overall survival than those of high grade (micropapillary/solid predominant/ colloid predominant/ invasive mucinous adenocarcinoma). These results suggested that histological subtyping could be considered as a powerful predictor of biological behavior in lung adenocarcinoma, patients with poor prognostic histological components, such as micropapillary or solid predominant, should receive more careful follow-up and be monitored more closely for disease progression. For those patients with unresectable lung cancer and those who do not wish to undergo surgery, biopsy specimens might be the only available material for histological subtyping. However, accurate assessment of adenocarcinoma histologic subtype on biopsy samples is rather challenging as small biopsy specimens may be limited in representing the entire tumor due to intra-tumor heterogeneity.

Imaging can provide good spatial resolution and capture information that assesses whole tumor heterogeneity [11]. With recent technological advance in medical imaging, computer-assisted image texture analysis which allows noninvasively quantifying tumor heterogeneity by extracting quantitative parameters of the tumor has shown promising results in tumor differentiation, outcomes prediction, and pathologic features characterization [12–16]. Computed tomography (CT) has been widely used as the initial radiologic modality for lung cancer patients, many researchers have attempted to incorporate CT texture analysis in clinical practice. In a recent study conducted by Chae et al. [17], computerized texture analysis of persistent part-solid ground-glass nodules has the potential ability to differentiate pre-invasive lesions from invasive pulmonary adenocarcinomas. Similarly, Son et al. [18]demonstrated that quantitative analysis of preoperative CT imaging metrics can help distinguish invasive adenocarcinoma from pre-invasive or minimally invasive adenocarcinoma in pulmonary ground-glass opacity nodules. To the best of our knowledge, there were relatively few studies that investigated the role of texture analysis on contrast-enhanced CT (CECT) in lung adenocarcinoma. In this study, we reviewed CECT images of 148 surgically resected lung adenocarcinoma patients, and investigated its correlation with pathologic grade as determined by new IASLC/ATS/ERS classification.

## RESULTS

Agreement between two radiologists tested by using *κ* coefficient analysis was perfect for enhancement heterogeneity (kappa = 0.807).

One hundred and forty-eight patients with 148 peripheral lung adenocarcinomas were included in this study. The clinicopathological characteristics were summarized in Table 1. The median age was 60 years, ranging from 30 to 76 years. Of the 148 patients, 81 (54.7%) were never smokers, and 67 (45.3%) were current or former smokers. The majority patients were female (59.2%), while 61 patients were male (41.2%). Lobectomy was performed in 140 patients, pneumonectomy in 1, wedge resection in 2, and segmentectomy in 5 patients. 100 (67.6%) patients were of early stage (I or II), and 48 (32.4%) with advanced stage (III or IV).

**Table 1:** Patient demographics

Variables	*N* (*n*= 148) (%)
Age, median (range)	60 (30-76)
Gender	
Male	61 (41.2)
Female	87 (59.2)
Smoking history	
Never smokers	81 (54.7)
Smokers	67 (45.3)
Stage	
I	86 (58.1)
II	14 (9.5)
III	40 (27.0)
IV	8 (5.4)
Histologic subtype	
acinar predominant	65 (43.9)
lepidic predominant	36 (24.3)
papillary predominant	12 (8.1)
micropapillary predominant	6 (4.1)
solid predominant	27 (18.2)
invasive mucinous adenocarcinoma	2 (1.4)

According to the new IASLC/ATS/ERS classification, the most frequent subtype in our cohort was acinar predominant (*n* = 65, 43.9%), 24.3% (*n* = 36) of the cases were lepidic predominant, 18.2% (*n* = 27) of the cases were solid predominant, 8.1% (*n* = 12) of the cases were papillary predominant, 4.1% (*n* = 6) of the cases were micropapillary predominant, and 1.4% (*n* = 2) of the cases were invasive mucinous adenocarcinoma.

Using our pre-defined groupings we identified adenocarcinoma *in situ* and minimally invasive adenocarcinoma as low grade; lepidic, acinar, and papillary predominant adenocarcinoma as intermediate grade; solid, micropapillary, colloid predominant, and invasive mucinous adenocarcinoma as high grade. No adenocarcinoma *in situ* or minimally invasive adenocarcinoma was found in this study; therefore two pathologic grade groups were identified in this study: intermediate grade (*n* = 113) and high grade (*n* = 35). Intermediate grade comprised of lepidic predominant, acinar predominant, and papillary predominant. High grade consisted of micropapillary predominant, solid predominant, and invasive mucinous adenocarcinoma.

The association between pathologic grade and clinical characteristics was showed in Table 2. There was no significant difference in mean age, gender, or stage between intermediate grade and high grade groups. Significantly higher incidence of intermediate pathologic grade was found in never smokers (84.0%) than smokers (67.2%) (*P* = 0.020).

**Table 2:** Association between clinical characteristics and pathologic grade

Clinical characteristics	Pathologic grade		*P* value
	Intermediate (*n* = 113)	High (*n* = 35)	
**Age, mean (range)**	58.06 (30 - 76)	59.43 (45 - 74)	0.332
**Gender, *N* (%)**			
Male	42 (68.9)	19 (31.1)	0.080
Female	71 (81.6)	16 (18.4)	
**Smoking history, *N* (%)**			
Never smokers	68 (84.0)	13 (16.0)	0.020
Smokers	45 (67.2)	22 (32.8)	
**Stage, *N* (%)**			
I or II	77 (77.0)	23 (23.0)	0.837
III or IV	36 (75.0)	12 (25.0)	

In intermediate pathologic grade group, 31 tumors demonstrated homogeneous enhancement, 82 tumors had heterogeneous enhancement; while in high pathologic grade group, 11 tumors showed homogeneous enhancement, and the other 24 tumors manifested as heterogeneous enhancement. The differences in homogeneous enhancement between the intermediate grade and high grade were not significant (26.2% *vs* 22.6%; *p* = 0.671). Among those 54 CT features extracted by 3D Slicer, 29 of them showed significant difference between intermediate and high pathologic grade groups (Table 3).

**Table 3a:** Comparison of CT features between intermediate grade group and high grade group (mean ± SD of each parameter, Student's t-test for the comparison)

CT features		Pathologic grade		*P* value
		Intermediate (*n* = 113)	High (*n* = 35)	
F17	Surface: Volume Ratio	0.702 ± 0.280	0.565 ± 0.260	0.011
F18	Compactness 1	13.106 ± 7.862	19.697 ± 13.236	0.008
F19	Compactness 2	0.133 ± 0.034	0.137 ± 0.030	0.538
F20	Maximum 3D Diameter	29.480 ± 11.435	33.541 ± 12.300	0.073
F22	Sphericity	0.507 ± 0.045	0.513 ± 0.040	0.481
F35	IMC1	−1.929 ± 0.090	−1.871 ± 0.101	0.002
F44	SRE	0.911 ± 0.020	0.913 ± 0.021	0.546
F45	LRE	3.890 ± 1.814	4.026 ± 1.425	0.686
F48	RP	0.936 ± 0.018	0.924 ± 0.022	0.001

**Table 3b:** Comparison of CT features between intermediate grade group and high grade group (mean rank of each parameter, Mann–Whitney U-test for the comparison)

CT features		Pathologic grade		*P* value
		Intermediate (*n*= 113)	High (*n*= 35)	
F1	Energy	72.63	80.54	0.340
F2	Entropy	69.19	91.66	0.007
F3	Minimum Intensity	72.31	81.56	0.265
F4	Maximum Intensity	76.66	67.53	0.271
F5	Mean Intensity	74.63	74.09	0.948
F6	Median Intensity	76.42	68.30	0.327
F7	Range	77.13	66.00	0.179
F8	Mean Deviation	79.48	58.43	0.011
F9	Root Mean Square	79.91	57.03	0.006
F10	Standard Deviation	79.60	58.03	0.009
F11	Skewness	72.02	82.51	0.206
F12	Kurtosis	76.21	68.97	0.383
F13	Variance	79.60	58.03	0.009
F14	Uniformity	68.91	92.54	0.004
F15	Volume cc	69.57	90.43	0.012
F16	Surface Area mm^2	69.79	89.71	0.016
F21	Spherical Disproportion	79.74	57.57	0.008
F23	Autocorrelation	72.35	81.46	0.272
F24	Cluster Prominence	75.23	72.14	0.710
F25	Cluster Shade	76.40	68.37	0.333
F26	Cluster Tendency	73.65	77.26	0.663
F27	Contrast	72.27	81.71	0.255
F28	Correlation	91.68	51.31	0.000
F29	Difference Entropy	69.21	91.57	0.007
F30	Dissimilarity	70.98	85.86	0.073
F31	Energy (GLCM)	69.26	91.43	0.008
F32	Entropy(GLCM)	79.88	57.11	0.006
F33	Homogeneity 1	68.88	92.63	0.004
F34	Homogeneity 2	68.80	92.91	0.004
F36	IDMN	69.54	90.51	0.011
F37	IDN	69.54	90.51	0.011
F38	Inverse Variance	68.81	92.86	0.004
F39	Maximum Probability	68.74	93.10	0.003
F40	Sum Average	71.15	85.31	0.088
F41	Sum Entropy	79.92	57.00	0.006
F42	Sum Variance	70.04	88.89	0.023
F43	Variance (GLCM)	72.35	81.43	0.274
F46	GLN	68.45	94.03	0.002
F47	RLN	69.64	90.20	0.013
F49	LGLRE	78.98	60.03	0.022
F50	HGLRE	76.13	69.23	0.405
F51	SRLGLE	79.37	58.77	0.013
F52	SRHGLE	76.20	69.00	0.385
F53	LRLGLE	73.73	77.00	0.693
F54	LRHGLE	74.99	72.91	0.802

**Figure 1:**
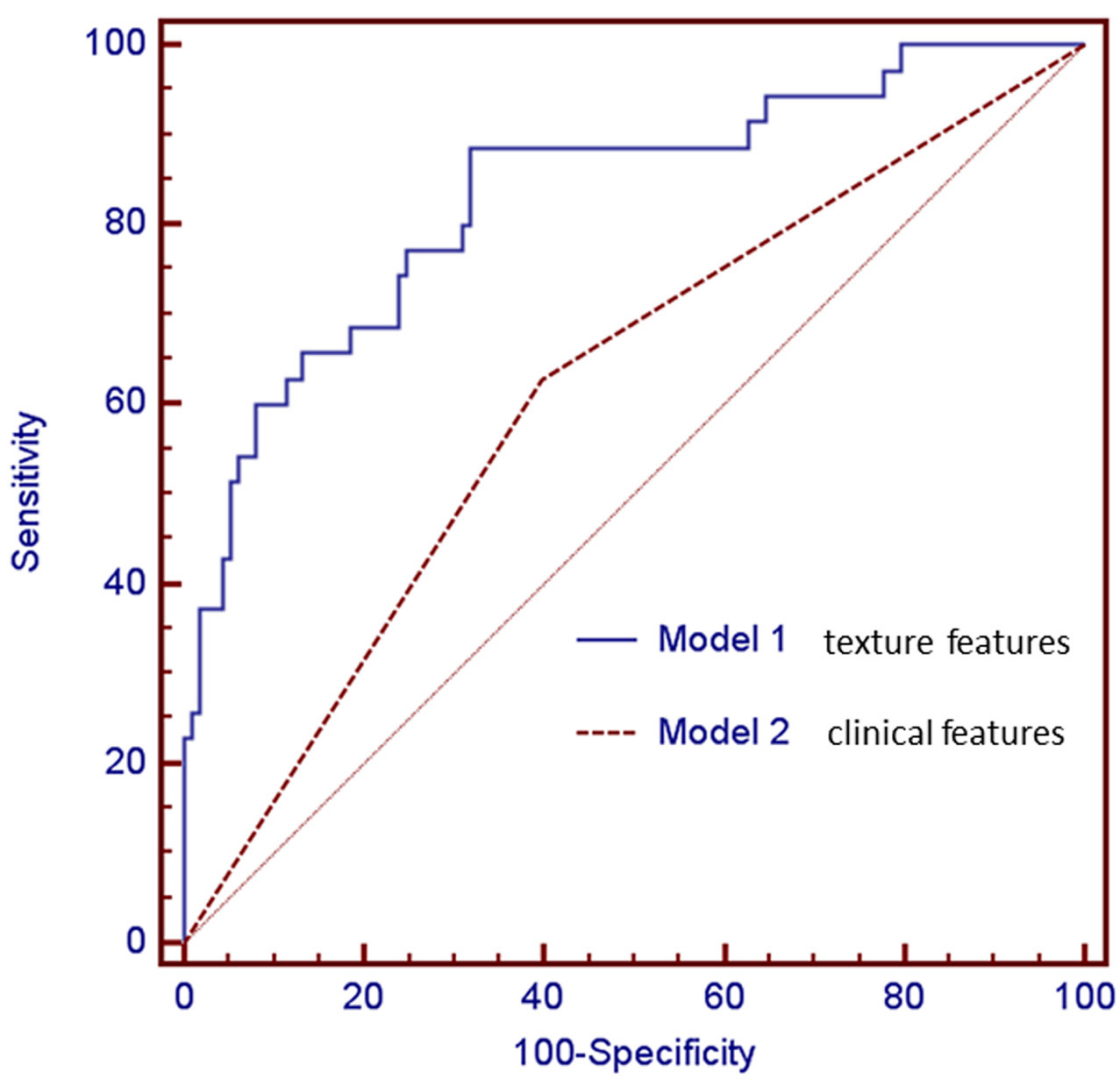
Comparisons of ROC curves of two logistic regression models derived from texture features (Model 1), and clinical feature (Model 2) for the prediction of pathologic grade The model generated with texture features (Model 1, AUC = 0.834) was superior to the model with clinical variable (Model 2, AUC = 0.615). There was significant difference in AUCs between these two models (*P* = 0.0001).

Multivariate logistic regression analysis revealed that (Table 4) smoking history was proved to be independent predictors of pathologic grade, compared with never smokers, smokers with peripheral lung adenocarcinoma had a statistically significant decrease in the incidence of being intermediate pathologic grade (OR, 0.391; 95% CI, 0.179 - 0.855; *P* = 0.019). The AUC was 0.615 (95% CI: 0.532 - 0.694; *P* = 0.015), and the sensitivity and specificity were 62.9% and 60.2%, respectively. For the model generated with texture features, F33 (Homogeneity 1) (OR, 1.393; 95% CI, 1.104 - 1.758; *P* = 0.005) and F38 (Inverse Variance) (OR, 0.825; 95% CI, 0.692 - 0.983; *P* = 0.032) were independent predictors of pathologic grade, and the AUC was 0.834 (95% CI: 0.764 - 0.890; *P* < 0.0001) with 88.6% sensitivity and 68.1% specificity. The higher AUC was achieved by texture feature model, and it was superior to the clinical model (*P* = 0.0001) (Figure 1).

**Table 4:** Multivariate logistic regression models for the differentiation of intermediate and high pathologic grade groups with clinical variables and texture features

Model	Features	Odds ratio (95% CI)	*P* value	AUC (95% CI)
Model1: Clinical variables	Smoking history (smokers VS. never smokers)	0.391 (0.179 - 0.855)	0.019	0.615 (0.532 - 0.694)
Model 2: Texture features	F33 (Homogeneity 1)	1.393 (1.104 - 1.758)	0.005	0.834 (0.764 - 0.890)
	F38 (Inverse Variance)	0.825 (0.692 - 0.983)	0.032	

## DISCUSSION

Tumors are heterogeneous on both the genetic and histopathological levels, with intratumoral spatial variation in the cellularity, angiogenesis, extravascular extracellular matrix, and areas of necrosis [19]. Intratumoral heterogeneity is a well-recognized feature of malignancy that reflects areas of high cell density, necrosis, hemorrhage, and myxoid change [20], and it is considered as an important prognostic factor since high intratumoral heterogeneity may be associated with higher tumor grade [21]. Texture analysis is an image processing algorithm that based on analyzing the distribution and relationship of pixel or voxel-gray levels in the image which is only qualitatively assessable by the human visual system to a limited degree [22]. This computer assisted technique has the potential to allow extraction of quantitative parameters related to tissue heterogeneity.

Recent successful application of texture analysis on contrast enhanced CT of NSCLC [15, 16] has reported that texture analysis could be predictor of survival and treatment response. Ravanelli et al. [16] found that in the adenocarcinoma group, the product of tumor uniformity and grey level (GL*U) extracted from CECT was the unique independent variable correlating with treatment response to first-line chemotherapy. This study was performed on a single 5-mm axial image through the largest cross-sectional area of the tumor. A study of 98 patients with unresectable NSCLCs that underwent definitive concomitant chemoradiotherapy [15] demonstrated that entropy, skewness, and mean attenuation were significantly associated with 3-year overall survival, and higher entropy (adjusted hazard ratio [HR],2.31; *P* = 0.040), higher skewness (adjusted HR,1.92; *P* = 0.046), and higher mean attenuation (adjusted HR,1.93; *P* = 0.028) were independent predictors of decreased 3-year overall survival. In contrast to the former study, regions of interest were drawn around the boundary of the whole tumor volume. Comparison between single slice 2D with 3D texture analysis was relatively rare, and the findings were controversial. In a study involving 55 patients with primary colorectal cancer, entropy was higher and uniformity lower for the whole tumor volume compared to the largest cross-sectional area at all filter levels, and Kaplan Meier analysis showed better separation of entropy and uniformity for whole tumor analysis for 5-year overall survival, indicating that whole tumor analysis appears more representative of tumor heterogeneity [23]. Nevertheless, Lubner et al. [12] demonstrated that comparison of 2D *vs*. 3D measurements of single lesions showed fairly comparable results in a subset of 20 patients with hepatic metastatic colorectal cancer. We assumed that the use of single slice rather than the whole tumor to extract features is a limitation given the overall aim to quantify heterogeneity, although it is less time consuming; meanwhile, subject selection of the largest cross section of the lesion may vary among different radiologists. Therefore, texture analysis was explored on the whole tumor volume in our study.

Several studies have confirmed that the new IASLC/ATS/ERS international multidisciplinary lung adenocarcinoma classification has significant prognostic and predictive value regarding death and recurrence [3, 24, 25]; for example, lepidic predominant pattern had a lower recurrence risk, whereas micropapillary and solid predominant patterns had a higher recurrence risk. Concerning the relationship between angiogenesis and CT contrast enhancement, studies on lung cancer demonstrate a positive correlation between enhancement values and microvessel density measured on histological specimens [26, 27]. Meanwhile, study assessing the prognostic potential of texture analysis showed that high tumor heterogeneity is associated with poorer outcome [28]. Thus, we postulate that heterogeneity measured on CECT which provides information about the grade of enhancement could have potential relationship with tumor biology. This hypothesis was confirmed by the fact that 29 texture features were significantly associated with pathologic grade, including 6 first-order statistics features, 5 morphology and shape features, 13 texture features (GLCM), and 5 texture features (GLRL). First-order statistics which is dependent on a single pixel value rather than its interaction with neighboring pixels relates to gray-level frequency distribution within the region of interest [29]. The co-occurrence measurements calculated using spatial gray-level dependence matrices determine how often a pixel of intensity i finds itself within a certain relationship to another pixel of intensity j; and the run-length matrix analyzes texture in a specific direction [19]. In contrast to computer assisted image analysis, diagnostically relevant heterogeneity perceived by the naked eye demonstrated no significant difference between intermediate and high grade group. This suggests that image interpretation based on a visual process could not fully describe the underlying biological heterogeneity.

Multivariable regression analyses showed that texture features of F33 (Homogeneity 1) and F38 (Inverse Variance) were independent predictors for pathologic grade, and this model has higher predictive power than the model with clinical variable (smoking history), since the AUC for the model with texture features (AUC = 0.834) was significantly greater than the clinical model (AUC = 0.615) (*P* = 0.0001). This interesting finding indicates that texture analysis as a non-invasive method of assessing the heterogeneity within a tumor might be of clinical benefit in predicting pathologic grade.

Our study has some limitations. First, this was a retrospective study with single institution design; therefore there might have been unavoidable selection bias. Second, the sample size was relatively small. In addition, since the histologic heterogeneity of lung adenocarcinoma limits the diagnostic accuracy of small biopsy or cytology specimens compared to resected specimens, only patients with surgically resected lung adenocarcinoma were included in the current study. Furthermore, the follow-up time after surgery was not sufficient to evaluate the relationship between texture features and survival. Finally, potential variables such as patients’ cardiac output and blood volume which might influence tumor enhancement were not taken into consideration.

In summary, the preliminary results obtained from our study indicated that texture analysis of CECT images in lung adenocarcinomas which provides objective measurements of heterogeneity may serve as an important imaging biomarker to predict pathologic grade as determined by new IASLC/ATS/ERS classification.

## MATERIALS AND METHODS

### Patients

This monocentric and retrospective study was approved by our institutional review board, and the need for informed consent was waived. Prior written informed consent had been acquired from patients with regard to the use of CT imaging. Between March 2013 and January 2014, a total of 213 patients were found in our surgical database that fulfilled the following criteria: (1) histopathologically confirmed lung adenocarcinoma using surgically resected specimens, (2) pre-operative thin-section CT examination of both unenhanced and contrast-enhanced at the same day, and (3) the location of the tumor was peripheral. Then, we excluded patients because of duration of CT examination and surgery exceeded one month (*n* = 11), preoperative adjuvant chemotherapy or radiotherapy (*n* = 15), poor image quality resulting from failure of breath holding during CT examination (*n* = 2), cases with multiple lesions on CT images that could not be conclusively correlated with the lesions documented in the pathology report (*n* = 1), or cases that contained ground glass opacity (GGO) component on CT images as ascertained by two radiologists (*n* = 36). Ultimately, 148 patients (61 men; 87 women; mean age, 58 years; range, 30-76 years) with pathologically proven lung adenocarcinoma comprised our study population. Clinical and pathologic information (age, gender, smoking history, stage, histologic subtype) were collected from the hospital's electronic medical records system. All tumors were restaged according to the TNM classification of malignant tumors (Union for International Cancer Control/American Joint Committee on Cancer, 7th edition) [30].

### CT examinations

Chest CT examinations were performed by using one of three MDCT systems: Somatom Sensation 64 (Siemens Medical Solutions, Forchheim, Germany), Light speed 16 (GE Medical Systems, Milwaukee, WI), and Discovery CT750 HD scanner (GE Medical Systems, Milwaukee, WI). For the 64-detector scanner, scanning parameters were as follows: 120 kVp with tube current adjusted automatically; pitch of 0.969; reconstruction thickness, 1.5 mm; reconstruction interval, 1.5 mm. For the 16-detector scanner and Discovery CT750 HD scanner, scanning parameters were as follows: tube voltage, 120 kVp; tube current, 150-200 mA; beam pitch, 0.969; reconstruction thickness, 1.25 mm; reconstruction interval, 1.25 mm. All of the patients underwent contrast-enhanced CT scan. Images were obtained after intravenous administration of 80 - 100 mL of non-ionic iodine contrast material (Ultravist, Bayer Pharma, Berlin, Germany; Omnipaque, GE Company, Shanghai, China) at a rate of 2.5 mL/sec using an automated injector. The CT scanning was performed with a 70-second delay. All CT scans were obtains in the supine position during breath holding at the end of full inspiration. Image resolution was determined by pixel spacing, and each case had the same image resolution.

### CT image analysis and post-processing

**Figure 2:**
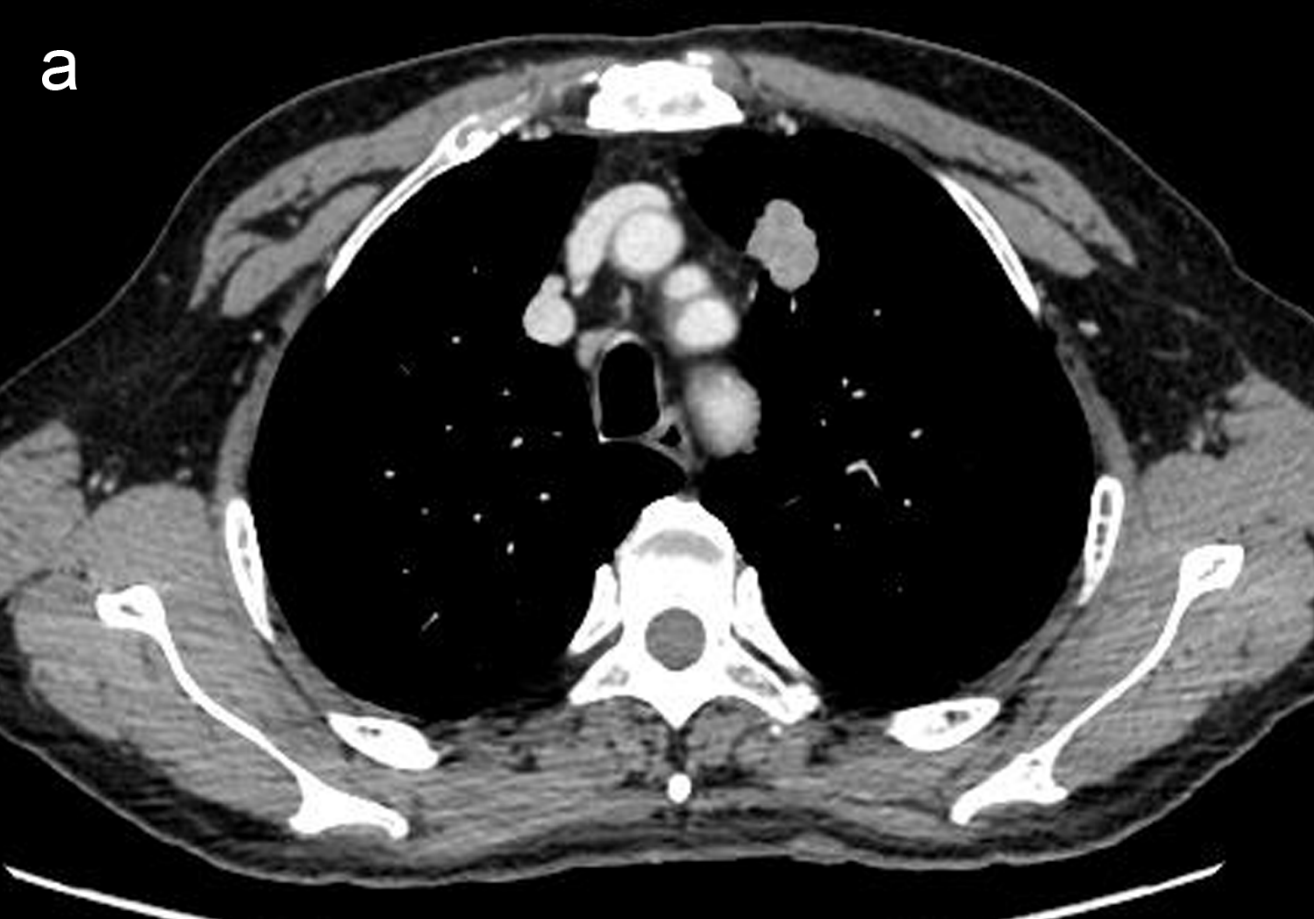

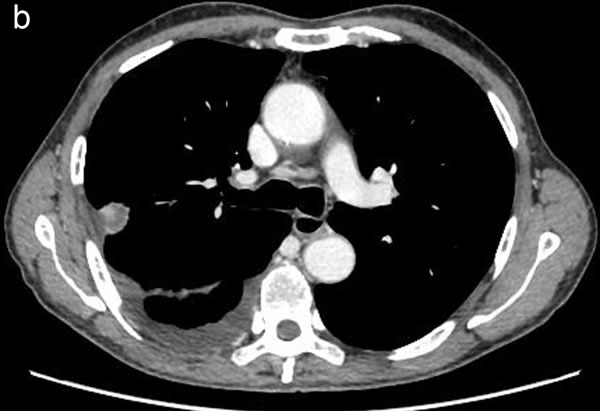
Representative CT images showing homogeneous enhancement (a) and heterogeneous enhancement (b)

**Figure 3:**
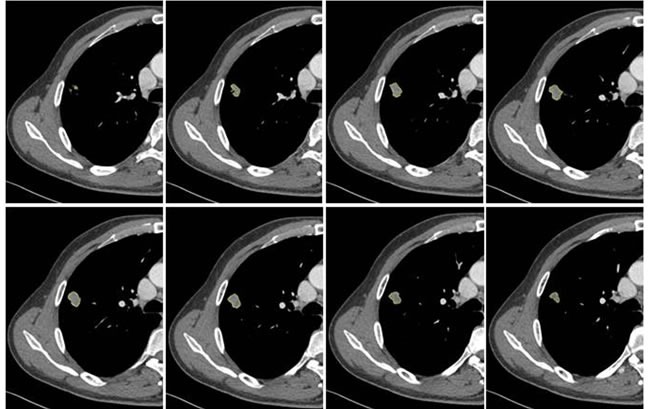

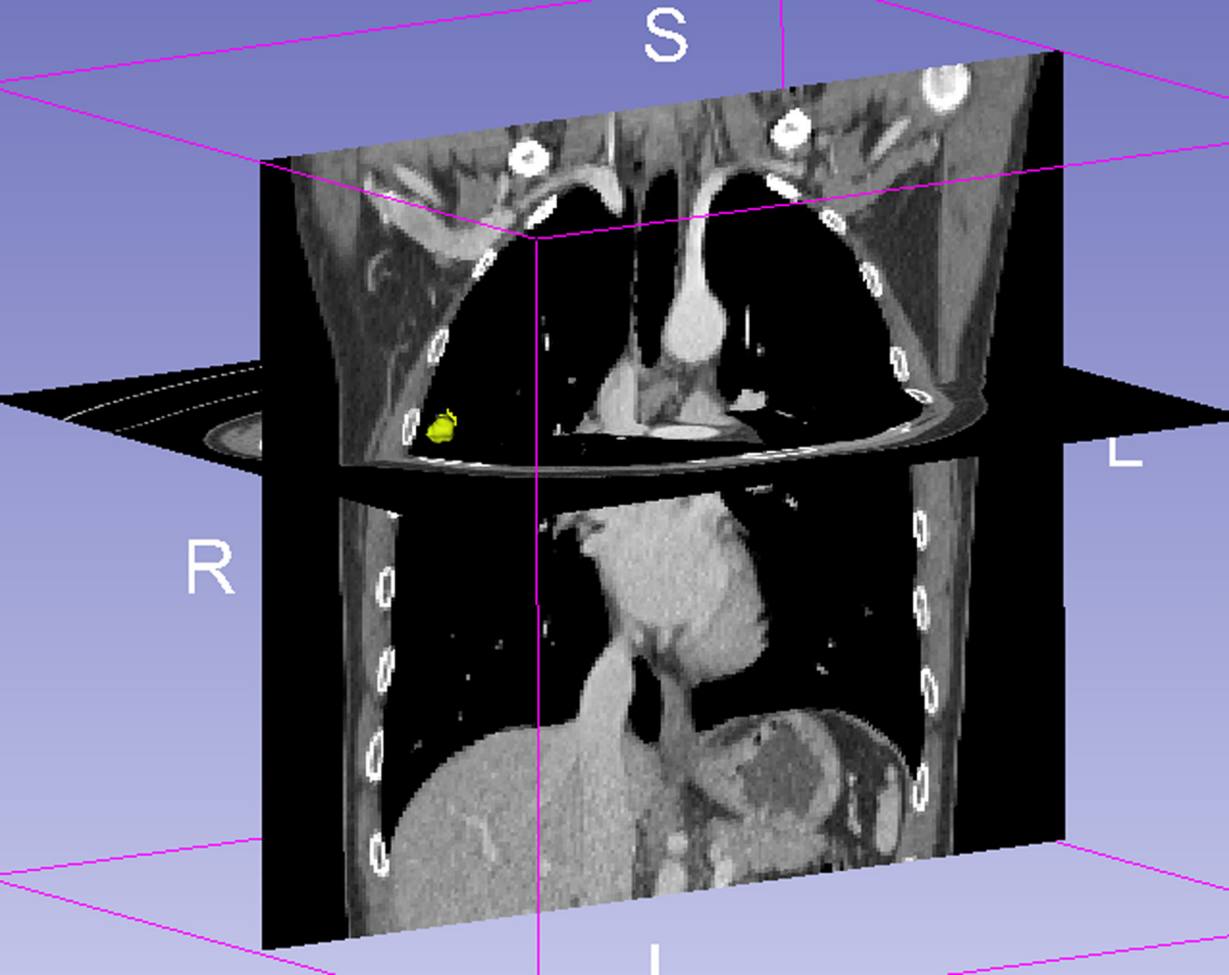
Example of CT images showing segmentation of lung tumor Semiautomatic tumor segmentation was done on every slice of the tumor using 3D slicer (a), and the 3D view of the segmented tumor (b) which was shown in yellow.

Two radiologists (with 6, and 3 years’ experience in thoracic CT imaging, respectively), who were blinded to clinical and histiopathological data, reviewed all CT images separately. They were asked to identify pulmonary lesions and to evaluate the enhancement heterogeneity according to the following criteria: homogeneous enhancement was defined as more than 90% of the tumor area was occupied by the same CT attenuation as ascertained by visual assessment; otherwise heterogeneous enhancement was considered (Figure 2). In case of disagreement, the third reviewer with 27 years of clinical experience in thoracic imaging, made the final decision. Then, semiautomatic tumor segmentation was done in consensus by three radiologists using a designated multi-platform, free and open source software package for visualization and medical image computing (3D slicer, version 4.4.0; available at: http://slicer.org/) (Figure 3). Totally, 54 features were extracted and they were divided into four categories (Supplementary Table 1, Image features metrics are available at: https://www.slicer.org/wiki/Documentation/Nightly/Modules/HeterogeneityCAD), including (1) First-Order Statistics, (2) Morphology and Shape, (3) Texture: GLCM, and (4) Texture: GLRL.

### Histologic diagnosis

All resected specimens were formalin fixed and stained with hematoxylin and eosin in the routine manner. As needed, mucin stains were performed to evaluate for mucin. All available hematoxylin and eosin-stained slides were reviewed by a pathologist. Histologic classification was done according to the proposed IASLC/ATS/ERS classification of lung adenocarcinomas; each tumor was reviewed using comprehensive histological subtyping. In the classification of invasive adenocarcinomas, 6 histologic patterns were defined as follows: (1) lepidic predominant; (2) acinar predominant; (3) papillary predominant; (4) micropapillary predominant; (5) solid predominant; and (6) variants of invasive adenocarcinomas. The predominant pattern is defined as the pattern with the largest percentage, not necessarily 50% or greater. When the individual pathologic diagnosis of a case did not coincide, agreement was obtained after discussion by both pathologists. Based on previous study [3], different subtypes were divided into three groups: low grade (adenocarcinoma *in situ* and minimally invasive adenocarcinoma), intermediate grade (lepidic, acinar, and papillary predominant adenocarcinomas), and high grade (solid, micropapillary, colloid predominant, and invasive mucinous adenocarcinoma).

### Statistical analysis

Statistical analyses were performed using SPSS Version 21.0 (IBM Corp. IBM SPSS Statistics for Windows) and MedCalc version 11.3.8.0 (MedCalc Software, Mariakerte, Belgium). Interobserver agreement was assessed using *κ* statistics. A kappa value of 0.81-1.00 was considered to indicate excellent agreement; 0.61-0.80, substantial agreement; 0.41- 0.60, moderate agreement; 0.21-0.40, fair agreement; and 0.00-0.20, poor agreement. Continuous variables were expressed as mean ± SD, and were compared using the Student's *t*-test or Mann-Whitney U-test; while categorical variables were presented as frequency, and were compared using the chi-square test or Fisher's exact test. Multiple logistic regression analysis was subsequently developed to determine the association between texture features and pathologic grade, and the results were expressed as an odds ratio (OR) with a 95% confidence interval (CI). In the multivariate logistic regression analysis, features with *p*-value of < 0.15 in univariable model were entered into the initial model. Backward elimination method was used to select the final predictive model; at each step, feature with *p*-value of > 0.05 was eliminated. Receiver operating characteristic (ROC) curve for the model was constructed and the area under the curve (AUC) was calculated. A *P* value of < 0.05 was considered to indicate a significant difference.

## SUPPLEMENTARY MATERIAL TABLE


