# Supplementary material for: Tumor heterogeneity assessed by texture analysis on contrast-enhanced CT in lung adenocarcinoma: association with pathologic grade
Source: Oncotarget. 2017 Feb 16;8(32):53664–74. doi: 10.18632/oncotarget.15399 (PMC5581139; doi:10.18632/oncotarget.15399)
Supplement: Supplementary file 1 [file oncotarget-08-53664-s001.pdf]

Tumor heterogeneity assessed by texture analysis on contrast-enhanced CT in lung adenocarcinoma: association with pathologic grade

Supplementary Material

Supplementary Table 1: Quantitative feature category

Feature category	Name of the feature			
First-Order Statistics	F1	Energy	F8	Mean Deviation
	F2	Entropy	F9	Root Mean Square
	F3	Minimum Intensity	F10	Standard Deviation
	F4	Maximum Intensity	F11	Skewness
	F5	Mean Intensity	F12	Kurtosis
	F6	Median Intensity	F13	Variance
	F7	Range	F14	Uniformity
Morphology and Shape	F15	Volume cc	F19	Compactness 2
	F16	Surface Area mm^2	F20	Maximum 3D Diameter
	F17	Surface: Volume Ratio	F21	Spherical Disproportion
	F18	Compactness 1	F22	Sphericity
Texture: GLCM	F23	Autocorrelation	F33	Homogeneity 1
	F24	Cluster Prominence	F34	Homogeneity 2
	F25	Cluster Shade	F35	IMC1
	F26	Cluster Tendency	F36	IDMN
	F27	Contrast	F37	IDN
	F28	Correlation	F38	Inverse Variance
	F29	Difference Entropy	F39	Maximum Probability
	F30	Dissimilarity	F40	Sum Average
	F31	Energy (GLCM)	F41	Sum Entropy
	F32	Entropy(GLCM)	F42	Sum Variance
			F43	Variance (GLCM)
Texture: GLRL	F44	SRE	F49	LGLRE
	F45	LRE	F50	HGLRE
	F46	GLN	F51	SRLGLE
	F47	RLN	F52	SRHGLE
	F48	RP	F53	LRLGLE
			F54	LRHGLE
